# Pretreatment and Thermal Stability of *Idesia polycarpa* Virgin Oil

**DOI:** 10.3390/foods14244210

**Published:** 2025-12-08

**Authors:** Hongxia Feng, Yazhen Zhao, Qingya Li, Wenhui Ye, Shuwen Zheng, Juncai Hou, Yunhe Chang

**Affiliations:** 1 School of Food Science and Engineering, Guiyang University, Guiyang 550005, China; fenghongxia914@163.com (H.F.); zhaoyazhen0509@163.com (Y.Z.); 19861627591@163.com (Q.L.); ywh485916143@163.com (W.Y.); houjuncai88@163.com (J.H.); 2 Engineering Technology Research Center for Processing and Comprehensive Utilization of Idesia Polycarpa of National Forestry and Grassland Administration, Guiyang 550005, China; 3 Guangdong Institute of Arts and Sciences, Zhanjiang 524400, China; 18730581931@163.com

**Keywords:** *Idesia polycarpa* oil, microwave vacuum drying, polar compounds, oil quality

## Abstract

As a novel woody oilseed, the application of *Idesia polycarpa* in oil processing has been limited by the lack of systematic research. In this study, the effects of different drying methods, impurity contents, and filtration cycles on the quality of *Idesia polycarpa* virgin oil were evaluated using physicochemical indicators such as acid value (AV), phospholipid content, color, etc. The thermal stability of the obtained *Idesia polycarpa* virgin oil was also assessed under high-temperature heating conditions in comparison with refined oil, soybean oil, and rapeseed oil. The results indicated that oil with higher transparency was obtained by microwave vacuum drying with zero impurity content and five filtration cycles with a peroxide value of 11.03 meq/kg and a phospholipid content of 1.07 mg/g. After high-temperature heating, lower total oxidation values (TOTOX = 106.05 ± 1.63) and polar compounds (7.99 ± 0.54 meq/kg) were detected in *Idesia polycarpa* virgin oil (IPVO) compared with soybean oil and rapeseed oil, confirming superior thermal stability. Overall, IPVO yield was improved and exceptional thermal stability was achieved by this pretreatment, underscoring its potential as a functional cooking oil for high-temperature culinary applications.

## 1. Introduction

*Idesia polycarpa Maxim* (*I. polycarpa*), commonly known as “oil grape,” [1,2] is a woody oil crop with high nutritional and economic value. Its fruits are rich in oil and contain a unique fatty acid profile, including unsaturated fatty acids (such as linoleic acid and oleic acid) [3,4] and natural antioxidants (e.g., polyphenols and tocopherols) [3,5]. These characteristics have recently made it a promising candidate for developing functional edible oils. Compared to conventional plant oils, cold-pressed *I. polycarpa* oil not only shows potential health benefits, such as regulating blood lipids and providing antioxidant effects [6], but also features a distinctive fruity aroma [7], enhancing its commercial appeal as a specialty oil. However, current research primarily focuses on analyzing its basic composition [8,9] and bioactive properties [10], while systematic studies on critical industrial processing parameters and oil stability remain limited. This gap significantly hinders the high-value utilization of *I. polycarpa* oil.

The quality and stability of virgin oil heavily depend on raw material pretreatment and processing techniques. Studies indicate that drying methods alter the fatty acid composition [11] and natural antioxidant composition of the oil, such as polyphenols, sterols, and carotenoids [12], thereby affecting the oxidative stability of the oil [13]. The impurity contents, i.e., mass ratio of stems to fruits, may influence oil color, acid value, and peroxide value by affecting raw material uniformity and impurity levels [14]. Meanwhile, filtration cycles—a critical step in refining—directly impacts residual colloids, phospholipids, and microparticles in the oil [15,16]. Collectively, these factors determine the sensory quality and shelf life of virgin oil [17]. However, the existing literature lacks quantitative research on processing parameters for *I. polycarpa* oil production, particularly regarding how process conditions of drying methods, impurity contents, and filtration cycles affect the quality of virgin oil. Thus, establishing scientifically controlled processing standards is urgently needed.

Furthermore, oils exposed to high temperatures not only undergo a decrease in chemical quality and potential safety hazards, but also experience sensory deterioration, including the development of undesirable flavors, off-odors, and darkening of color. These changes are closely related to oxidative and thermal degradation processes, as reported by previous studies using *Humulus lupulus* and *Origanum vulgare* essential oils to protect oils during deep-frying [18]. Under heating, oils easily undergo hydrolysis, oxidation, and polymerization, generating harmful substances like polar compounds and trans fatty acids [19]. Given that the composition of fatty acids plays a decisive role in the thermal degradation behavior of oils and fats—with oils rich in monounsaturated fatty acids exhibiting greater stability—the high proportion of polyunsaturated fatty acids in *I. polycarpa* oil may exacerbate such reactions [20]. Although studies have explored the thermal stability of traditional oils such as olive oil and rapeseed oil [21], the lipid transformation patterns and quality degradation mechanisms of *I. polycarpa* virgin oil (IPVO) under high-temperature heating processes remain unclear. Thus, systematically investigating how processing parameters influence its basic quality and evaluating its physicochemical changes during heating will provide essential insights for standardizing production and assessing its suitability for high-temperature cooking. These efforts will bridge the gap between laboratory research and the industrial-scale application of this oil.

## 2. Materials and Methods

### 2.1. Materials and Reagents

*I. polycarpa* fruits (Mianyang, China) were purchased from an *I. polycarpa* plantation in Mianyang, Sichuan, China. Fresh and ripe *I. polycarpa* fruits were selected, and after removing impurities and large branches and stems, the fruits were refrigerated at −20 °C for further experiment. Soybean oil and rapeseed oil were purchased from a local market (Guiyang China) and stored in a dark and dry place; 37 fatty acid methyl ester mixes and trans fatty acid methyl ester mixes were purchased from Shanghai Amperex Standard Technology Service Co. (Shanghai, China) Phenolphthalein, isopropanol, petroleum ether, glacial acetic acid, iso-octane, anhydrous sodium carbonate, and soluble starch were purchased from Tianjin Yongda Chemical Reagent Co. (Tianjin, China). p-Methoxyaniline was purchased from Shanghai Een Chemical Technology Co. (Shanghai, China).

### 2.2. Sample Processing

In order to study the effect of pretreatment on the quality of IPVO, the effects of drying methods, impurity contents and filtration cycles on the yield and quality of IPVO were mainly investigated, and the specific experimental processing conditions are shown in Table 1.

#### 2.2.1. Drying Methods

Fresh *I. polycarpa* fruits were subjected to drying using four distinct methods: MVD, MD, FIOD, and ASHPD. The drying parameters were determined based on pre-experiments, following the method outlined in the previous reports [22,23] with slight modifications, to achieve a moisture content of 3–4% in the dried product, indicating the completion of the drying process. Each drying method was performed in triplicate.

For the MVD method, the fresh *I. polycarpa* were evenly spread on a material tray inside a WBZ-100X microwave drying vacuum (Guiyang Xinqi Microwave Industry Co., Ltd., Guiyang, China). The vacuum pressure was maintained at 0.06 Mpa. The drying process was conducted in stages: initially at a microwave power of 36 kW for 100 min, followed by a fourth stage at 18 kW for 20 min, and a fifth stage at 9 kW for 20 min. The total drying time for MVD was 140 min.

In terms of the MD method, the fresh fruit of *I. polycarpa* on a baking tray at a temperature of 85 °C, utilizing a microwave drying apparatus (SAM-255, Pei’an Co., Ltd., Beijing, China) for a duration of 30 h. The microwave power settings were consistent with those employed in microwave vacuum drying.

For the FIOD method, the fresh fruits of *I. polycarpa* were arranged in a single layer on a baking tray and subjected to drying at a constant temperature of 60 °C using an infrared drying oven (model 766B-3, Shanghai Pudong Rongfeng Scientific Instrument Co., Ltd., Shanghai, China) until the moisture content of the dried fruit reaches 3% to 4%.

As for the ASHPD method, the fresh fruits of *I. polycarpa* were arranged flat on a baking tray and subjected to a consistent temperature in an air-energy heat pump drying oven (LAD-030, Guangdong Ruixing New Energy Technology Co., Ltd., Heshan, China) set at 60 °C for a duration of 20 h.

Then, the dried *I. polycarpa* fruits are fed into a CZR 309 oil press machine (Guangzhou Dehaiwei Industrial Equipment Co., Guangzhou, China) to prepare oil samples, at which time the impurity content was 0 and the filtration cycles were 1.

#### 2.2.2. Impurity Contents

After comparing the four drying methods (MVD, MD, FIOD, and ASHPD), we found MVD consistently produced higher oil yield and superior quality indices, including lower acid and higher clarity. Therefore, MVD was selected as the baseline drying method for the subsequent evaluation of impurity content and filtration cycles. Under the condition of microwave vacuum drying with a filtration cycle of one time, the effects of impurity contents of 0%, 10%, and 20% (mass ratio of stems to fruits) on the oil yield and physicochemical indices of IPVO were examined.

#### 2.2.3. Filtration Cycles

Under the condition of vacuum microwave drying treatment with zero impurity content, the effects of filtration cycles 1, 3 and 5 on the oil yield and physicochemical indexes of IPVO were investigated. Filtration of the oil was performed using a laboratory vacuum filtration apparatus equipped with a Buchner funnel and a vacuum flask. Qualitative filter paper was used for all treatments. Each cycle was carried out at ambient temperature to minimize oxidation, and the filtrate was collected immediately in amber glass bottles for subsequent analyses.

### 2.3. Preparation of I. polycarpa Oil

Based on the above preprocessing results and the method described in our laboratory, IPVO and *I. polycarpa* refined oil (IPRO) were prepared. A certain amount of dried *I. polycarpa* fruits, after careful selection, removal of impurities, drying, and de-stalking, were taken separately, and the oil press (CZR 309, Guangzhou Dehaiwei Industrial Equipment Co., Ltd., Guangzhou, China) was set to 105 °C for 20 min preheating and then pressed at the same temperature, and the IPVO was obtained by filtration cycles. Use enzymatic hydrolysis to remove colloidal impurities such as phospholipids and proteins from the obtained IPVO; Use alkaline solution to remove acid, reduce acid value, and improve oxidation stability; Afterwards, add activated clay for decolorization and stir at 94 °C for 94 min to adsorb pigments from the oil; Finally, IPRO was obtained by deodorization at 185 °C for 100 min [24].

### 2.4. Determination of Oil Yield and Physicochemical Indices of Oil Samples

Oil yield was determined according to the method of [25], the oil yield is calculated by the mass of oil obtained through cold pressing relative to the dry weight of the raw material, and the result is expressed in%. Acid value, color (L*, a*, and b*), moisture and volatiles and insoluble impurities were determined according to the method of Song et al. [26].The peroxide value, phospholipid content, and smoke point were determined, respectively, following Zhou et al. [27], Yang et al. [28], and Jafari, A. et al. [29] Transparency, odor, and flavor are based on GB/T 5525-2008 [30]. LS/T 3258-2018 Idesia polycarpa oil provides a basis for assessing whether oil samples meets quality standards [31].

### 2.5. Heating Test

Referring to the test method [32] for the heating test, the specific operation steps were as follows: 100 mL of *IPVO* was loaded into the heating bottle, and at the same time, *IPRO*, soybean oil (SO), and rapeseed oil (RO) were used as controls, and heated in a temperature-controlled oven (202-1AB, Tianjin Tester Instrument Co., Ltd., Tianjin, China) at 180 °C, 200 °C, and 220 °C for 2, 4, 6, 8, and 10 h. The primary objective of this study was to evaluate the long-term thermal stability and degradation behavior of the oils under accelerated heating conditions. Each treatment was carried out in triplicate. After heating, the oil samples were collected and stored in a refrigerator at −18 °C.

### 2.6. Determination of the Quality of Heated Fats and Oils

Acid value, peroxide value and anisidine value were calculated using the method described by Zhou et al. [27], and polar compounds were analyzed according to the method described by Jiang et al. [25]. The Total Oxidation (TOTOX) value was calculated according to the method described by Ahmadi, N. et al. [33], based on the peroxide value (PV) and anisidine value (AV), as shown in Formula (1). The formation of conjugated dienes (CD) and conjugated triene hydroperoxides (CT) in lipids was determined following the method of Oil samples were dissolved in isooctane to prepare a 1.0% (*w*/*v*) solution and mixed thoroughly. Absorbance was measured at 232 nm for CD and 268 nm for CT using a UV–Vis spectrophotometer with isooctane as the blank (i3, Jinan Haineng Instrument Co., Ltd., Jinan, China). The results were expressed as specific extinction values (K232 and K268) [34]. The concentrations of trans fatty acids were determined according to the standards of the People’s Republic of China. The fatty acid composition analysis was performed using an Agilent 8890 gas chromatograph (GC) system (8890, Agilent Technologies Ltd., Santa Clara, CA, USA) as described by [35]. Gas chromatography conditions: The gas chromatography column is a SH-Rt-2560 capillary column (100 m × 0.25 mm × 0.20 μm), the detector is a FID detector, and the temperature is programmed to increase from an initial temperature of 130 °C for 5 min. The temperature is then raised to 240 °C at a rate of 4 °C/min and maintained for 20 min. Due to limited sample availability and instrument scheduling constraints during the original experiment, fatty acids were measured only once.

(1) TOTOX=2PV+AV

### 2.7. Statistical Analysis of Data

All the indexes in this experiment were repeated three times, and the results were retained in two decimal places and expressed as ‘mean ± standard deviation. Origin 2021 was used for plotting and one-way analysis of variance (ANOVA) in SPSS V21.0 Statistics V21.0 was used for data analysis. Duncan’s multiple range test was used to determine the significant differences (*p* < 0.05) between treatments.

## 3. Results and Discussion

### 3.1. Effect of Different Pretreatments on the Oil Yield of I. polycarpa Virgin Oil

**Figure 1:**
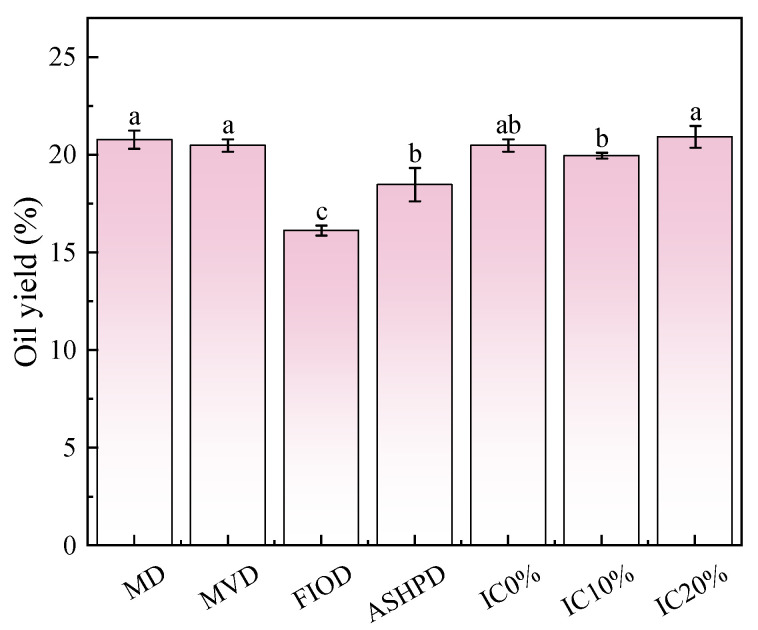
The effect of different pretreatments on the oil yield of *I. polycarpa* pressings. MD is Microwave Drying, MVD is Microwave Vacuum Drying, FIOD is Far-infrared Oven Drying, ASHPD is Air Source Heat Pump Drying, IC is Impurity Contents. Different letters show statistically significant differences (*p* < 0.05).

The oil yield of IPVO was significantly affected by pretreatment conditions, particularly drying methods and impurity content. As shown in Table S1 and Figure 1, MVD produced the highest oil yield, while FIOD resulted in the lowest. The enhanced yield under MVD is likely due to the rapid and uniform moisture removal, which promotes cell wall rupture and facilitates oil release. This mechanism aligns with previous findings that thermal treatments can improve oil extraction by disrupting cell structures [36]. Conversely, prolonged exposure to moderate temperatures in FIOD and ASHPD may induce surface hardening or crust formation, which impedes moisture escape and oil diffusion [37]. This effect may reduce oil recovery despite minimal thermal damage to sensitive components. It can thus be concluded that MVD and MD processes yield higher oil extraction rates for dried fruits, enabling maximized economic benefits.

Regarding impurity content, the results revealed a non-linear trend. At moderate impurity levels, oil yield increased. This may be attributed to the structural reinforcement provided by fibrous impurities, which increases mechanical resistance during pressing, thereby enhancing shearing and oil release [38]. However, excessive impurities can negatively affect oil quality and may pose challenges for downstream purification. When the impurity content is 0% and 20%, the effect on oil yield is not significant, which may be due to the macroscopic pores formed by the stem fibers improving the permeability of the pressed cake and reducing the residual oil in the cake. This result is consistent with the research of Mohamed Koubaa et al. [39]. In summary, the oil yield of *I. polycarpa* fruits treated with different impurity levels shows little variation. Statistical analysis confirmed that both drying method and impurity level significantly influenced oil yield (*p* < 0.01), with a high determination coefficient (R^2^ = 0.946), indicating strong model fit. MVD combined with appropriate impurity control appears optimal for maximizing IPVO yield without compromising processing efficiency or quality.

### 3.2. Effect of Different Pretreatments on the Quality of Idesia polycarpa Virgin Oil

#### 3.2.1. Effect of Different Drying Methods on the Quality of *Idesia polycarpa* Virgin Oil

Drying methods significantly influenced the physicochemical characteristics of IPVO. As shown in Table 2, MVD produced oils with higher brightness, lower acid and peroxide values, and better clarity than MD, FIOD, and ASHPD. Similarly, scholars have found that microwave-assisted hot air drying of corn can effectively preserve color and carotenoids while reducing fatty acidity and maintaining corn quality [40]. These results suggest that MVD effectively reduces oxidation and thermal degradation by limiting exposure to oxygen and high temperatures, preserving natural pigments and antioxidant compounds. In contrast, FIOD and ASHPD resulted in lower brightness values and darker oil appearance, likely due to prolonged heat exposure that promotes pigment degradation and lipid oxidation. Furthermore, oils from these methods exhibited higher phospholipid content, possibly due to less efficient removal or degradation of membrane-derived phospholipids under moderate drying temperatures and extended drying times, which is known to reduce oil clarity and promote turbidity [41]. Elevated phospholipid levels can also decrease the smoke point and contribute to bitter or burnt flavors, reducing the sensory quality of the oil.

The acid and peroxide values of IPVO from MVD and MD were significantly lower than those from FIOD and ASHPD, indicating that MVD and MD better preserve oil stability. This may be due to the more controlled thermal profiles in MVD and MD, which reduce oxygen contact and thermal stress. Statistical analysis of the physicochemical indicators also reveals that the first-press oil extracted from fresh *I. polycarpa* after microwave vacuum drying exhibits numerically lower physicochemical values compared to the other three drying methods.

As shown in Table 2, compared with the LS/T 3258-2018 industry standard for *I. polycarpa* oil, all relevant indicators of *I. polycarpa* oil meet the specified limit standards. Therefore, after pressing and extraction of *I. polycarpa* nuts, IPVO is obtained. Through degumming, neutralization, decoloring, and deodorization, qualified refined IPRO can be obtained. Furthermore, a 90-day subchronic toxicity study conducted by Zeng et al. according to OECD guidelines further confirmed its edible safety: even at high doses, the oil exhibited no toxic effects in experimental rats, and a no-observed-adverse-effect level (NOAEL) was established [2]. Collectively, these findings indicate that *I. polycarpa* oil represents a potential vegetable oil resource that complies with industry standards and offers assured safety.

#### 3.2.2. The Effect of Different Impurity Content on the Quality of *Idesia polycarpa* Virgin Oil

Impurity content plays a crucial role in determining the physicochemical stability and visual quality of virgin oils. As shown in Table 3, increasing impurity levels in IPVO led to reduced brightness and clarity, along with significant increases in acid value, peroxide value, and phospholipid content. The presence of impurities—primarily from fruit stalks and residual plant material—can increase the moisture content and introduce catalytic metal ions, accelerating hydrolysis and oxidation reactions. Oils with high impurity content are more prone to rancidity and have reduced shelf life due to elevated levels of free fatty acids and peroxides [37]. The rising phospholipid levels observed with higher impurities also contribute to emulsion formation and turbidity, further reducing consumer acceptability.

Although moderate impurity content may improve oil yield by enhancing mechanical friction during pressing, it poses a trade-off with oil stability and clarity. Therefore, controlling impurity levels is essential to ensuring oil quality. The data indicate that IPVO with low impurity levels presents better oxidative stability and appearance. These results emphasize the importance of thorough de-stemming and raw material screening prior to oil pressing. Efficient removal of fruit stalks and other fibrous materials not only improves oil clarity but also minimizes oxidative degradation and off-flavor development, ultimately enhancing product quality.

#### 3.2.3. The Effect of Different Filtering Times on the Quality of *Idesia polycarpa* Virgin Oil

Filtration plays a key role in refining virgin oil by removing suspended solids, phospholipids, and other colloidal particles. As shown in Table 4, increasing the number of filtration cycles led to improved clarity and reduced insoluble impurities in IPVO. The oil became visually more transparent, and key indicators such as turbidity and sediment content declined significantly with successive filtrations. This trend aligns with the understanding that multiple filtrations enhance the removal of fine particulates, including phospholipids, pigments, and oxidized colloids [42]. As a result, the refined oil exhibits improved sensory attributes and stability. However, it was also observed that excessive filtration slightly increased acid value and moisture content, which may be attributed to prolonged exposure to air and humidity during extended processing [43].

Therefore, while filtration improves appearance, over-filtration may compromise certain quality parameters. The optimal filtration cycle appears to balance effective impurity removal with minimal exposure to oxidative conditions. These findings support adopting a moderate number of filtration cycles—such as three to five—to achieve both clarity and oxidative stability. Proper filtration strategy contributes significantly to the commercial appeal and shelf-life extension of IPVO.

Overall, the results indicated that the pretreatment conditions had a pronounced influence on both oil yield and quality characteristics of IPVO. Among the four drying methods, MVD not only ensured a high oil yield but also maintained superior quality indices, such as lower acid and peroxide values and higher transparency. Moreover, when the impurity content was reduced to 0% and the oil was subjected to five filtration cycles, the MVD-pretreated oil showed further decreases in phospholipids and insoluble impurities. These findings suggest that the combination of MVD drying, 0% impurity content, and five filtration cycles represents the pretreatment condition for obtaining high-quality IPVO.

### 3.3. Changes in the Physicochemical Indices of IPVO, IPRO, SO and RO During the Heating Process

#### 3.3.1. Acid Value

**Figure 2:**
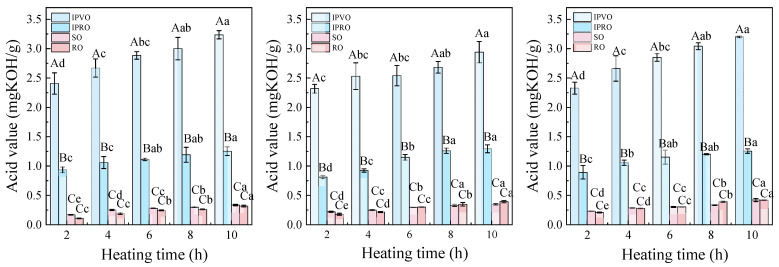
Changes in acid value of IPVO, IPRO, SO and RO were observed at different heating times (2, 4, 6, 8, and 10 h) during heating at 180 °C (**left**), 200 °C (**middle**), and 220 °C (**right**). Different uppercase letters indicate significant differences (*p* < 0.05) between different oil samples at the same heating time and given temperature, while different lowercase letters indicate significant differences (*p* < 0.05) between different heating times for the same oil sample at the same temperature.

The acid value (AV) serves as a vital indicator for assessing the hydrolytic degradation of oils and reflects the concentration of free fatty acids (FFAs) formed from the breakdown of triglycerides [44]. As shown in Figure 2, the acid values of all four oils—IPVO, IPRO, SO and RO—increased with rising temperature and prolonged heating time. Specifically, after 10 h of heating at 220 °C, the AV of IPVO rose to 0.88 mg/g, representing a 37.60% increase compared to its initial value, whereas IPRO, SO, and RO showed increases of 40.32%, 82.33%, and 101.25%, respectively.

Interestingly, although IPVO maintained a relatively high acid value at all time points due to its unrefined nature, the rate of increase was consistently lower than that observed in SO and RO. This increase indicates that prolonged exposure to elevated temperature promotes lipid hydrolysis, leading to the accumulation of free fatty acids, which may negatively affect both the flavor and stability of the oil. However, it also suggests that the natural antioxidants retained in IPVO, including tocopherols, phytosterols, and squalene, play a crucial role in mitigating thermal hydrolysis of triglycerides [45]. In contrast, RO exhibited the highest acid value increase, which may be attributed to its high unsaturated fatty acid content and lack of protective minor components, making it more susceptible to both oxidative and hydrolytic degradation [46]. Compared with SO and RO, IPRO has a higher acid value, indicating that laboratory scale refining processes are not entirely effective in removing free fatty acids compared to industrial refining practices.

These findings are supported by prior research, which indicates that oils with a higher degree of unsaturation are more prone to degradation under thermal conditions [44]. Additionally, antioxidant supplementation has been shown to significantly inhibit the increase in acid value during frying [47]. Overall, the relatively stable acid profile of IPVO under thermal stress suggests its better suitability for high-temperature food processing applications compared to conventional refined oils.

#### 3.3.2. Peroxide Value

**Figure 3:**
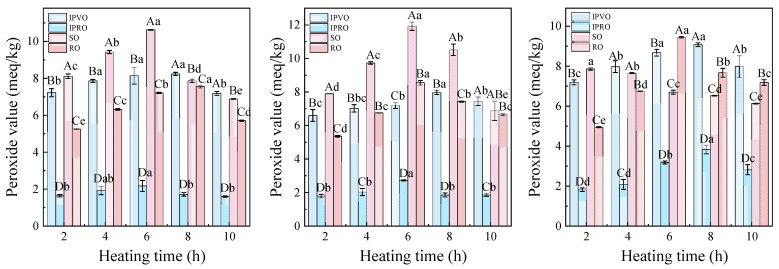
Changes in peroxide value of IPVO, IPRO, SO and RO were observed at different heating times (2, 4, 6, 8, and 10 h) during heating at 180 °C (**left**), 200 °C (**middle**), and 220 °C (**right**). Different uppercase letters indicate significant differences (*p* < 0.05) between different oil samples at the same heating time and given temperature, while different lowercase letters indicate significant differences (*p* < 0.05) between different heating times for the same oil sample at the same temperature.

The peroxide value (PV) is commonly used to evaluate the extent of primary oxidation in oils by quantifying the hydroperoxides formed during the early stages of lipid peroxidation [48]. In this study, the PVs of IPVO, IPRO, SO, and RO increased rapidly in the initial phase of heating, followed by a gradual decline, showing a characteristic rise–fall pattern (Figure 3). At 180 °C, IPVO exhibited the most dramatic increase—up to 358.45% within the first two hours—indicating vigorous formation of hydroperoxides. By comparison, SO, RO, and IPRO increased by 164.15%, 80.33%, and 109.00%, respectively. Under the highest temperature condition (220 °C), the PVs of IPVO and SO peaked at around 8 h, reaching 7.2 meq/kg and 7.9 meq/kg, respectively, whereas RO and IPRO reached their peaks earlier at 6 h. The subsequent decline in PV is likely due to the decomposition of unstable hydroperoxides into aldehydes, ketones, and other secondary oxidation products [49]. This result is consistent with [50], who demonstrated that hydroperoxides are thermally labile and undergo secondary transformations under prolonged heating.

Interestingly, IPVO, despite its high initial oxidation rate, demonstrated a stable trend in the later stages of heating, implying a protective mechanism against the propagation of oxidative reactions. This may be attributed to the presence of phenolic compounds and tocopherols, which act as chain-breaking antioxidants. Additionally, the delayed peak in PV compared to RO and IPRO suggests that IPVO maintains oxidative stability over longer heating durations. These characteristics enhance the suitability of IPVO for culinary processes involving repeated or prolonged exposure to heat.

#### 3.3.3. Anisidine Value

**Figure 4:**
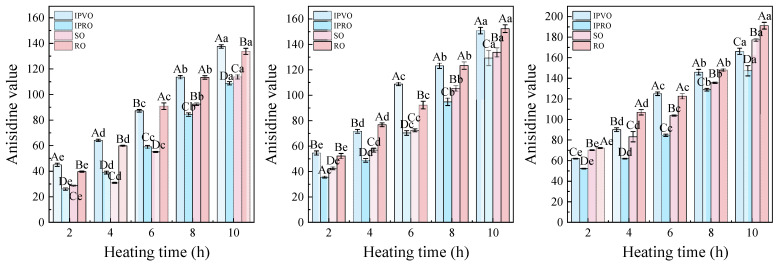
Changes in anisidine value of IPVO, IPRO, SO and RO were observed at different heating times (2, 4, 6, 8, and 10 h) during heating at 180 °C (**left**), 200 °C (**middle**), and 220 °C (**right**). Different uppercase letters indicate significant differences (*p* < 0.05) between different oil samples at the same heating time and given temperature, while different lowercase letters indicate significant differences (*p* < 0.05) between different heating times for the same oil sample at the same temperature.

The anisidine value (AV) is a critical marker for evaluating the accumulation of secondary oxidation products, particularly aldehydic compounds generated from the thermal decomposition of hydroperoxides in oils and fats [51]. Before heating, the Anisidine values of the IPVO, IPRO, SO, and RO were relatively low (0.12, 3.53, 0.98, and 0.01, respectively), indicating minimal aldehyde presence and the absence of secondary oxidation [52]. However, with increased heating temperature and time, all oils exhibited substantial rises in AV (Figure 4). After 10 h at 180 °C, the AV of IPVO surged to 137.57, exceeding that of SO (113.74) and IPRO (108.78), and closely approaching RO (133.80). At 200 °C, values increased further, with RO exhibiting the highest at 191.11, followed by SO (177.25), IPVO (166.24), and IPRO (147.36). This progressive trend underscores the intensification of oxidative degradation under elevated thermal stress. The relatively high increase in IPVO may stem from its rich linoleic acid content, which is more prone to forming peroxides and subsequent aldehydes [53]. Despite the high polyunsaturated fatty acid (PUFA) content, the lower AV in IPVO compared to RO suggests that its intrinsic antioxidants—such as tocopherols, phytosterols, and polyphenols—help mitigate the accumulation of secondary oxidation products. Similar protective effects were observed by [54], who demonstrated that thermal degradation can be moderated by the antioxidant profile and fatty acid composition.

Collectively, these results highlight the need to consider both lipid composition and antioxidant retention when evaluating thermal stability. While IPVO experiences an inevitable rise in AV under high-temperature conditions, its oxidative degradation is relatively restrained compared to traditional oils, reinforcing its potential as a heat-stable functional oil.

#### 3.3.4. Total Oxidation Value

**Figure 5:**
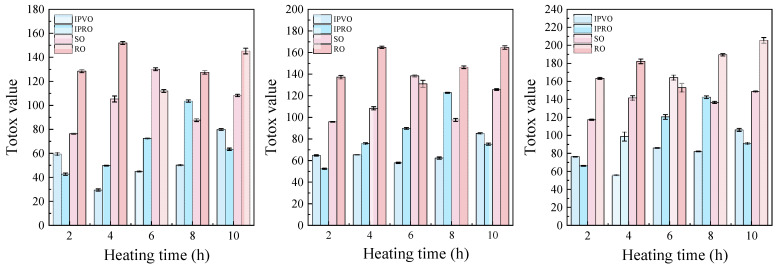
Changes in totox value of IPVO, IPRO, SO and RO were observed at different heating times (2, 4, 6, 8, and 10 h) during heating at 180 °C (**left**), 200 °C (**middle**), and 220 °C (**right**).

TOTOX Value (TV) aims to integrate the stages of primary and secondary oxidation by combining the determination of Peroxide Value and Anisidine Value in order to have an integrated view of the oxidation process [55]. As shown in Figure 5, the TV of all four oils (IPVO, IPRO, SO, and RO) increased progressively with prolonged heating time (2–10 h), indicating accelerated lipid oxidation under thermal stress. These results are consistent with previous research on perah seed oil, where the TOTOX value was also found to increase significantly with prolonged heating at frying temperature (170 °C) [56]. Among them, RO exhibited the highest TV throughout the heating process, suggesting its relatively poor oxidative stability under high-temperature conditions. SO also showed a marked increase in TV, reaching values close to those of RO at later heating stages. In contrast, IPVO and IPRO maintained lower TVs, particularly in the early heating period (2–6 h), demonstrating their superior resistance to thermal oxidation. Notably, IPVO exhibited a more gradual increase in TV compared to SO and RO, suggesting that trace components such as tocopherols and polyphenols scavenge peroxy radicals and carbonyl intermediates while simultaneously inhibiting the accumulation of PV and secondary oxidation represented by AV. Similarly to findings from studies on sunflower oil enriched with green tea extract [32], the relatively slow increase in total phenolic compounds (TV) in IPVO may be attributed to its inherent trace components—such as tocopherols and polyphenols—which help delay lipid oxidation. This indicates that IPVO possesses good thermal stability, making it suitable for cooking scenarios requiring moderate heating.

#### 3.3.5. Conjugated Dienes and Conjugated Trienes

**Figure 6:**
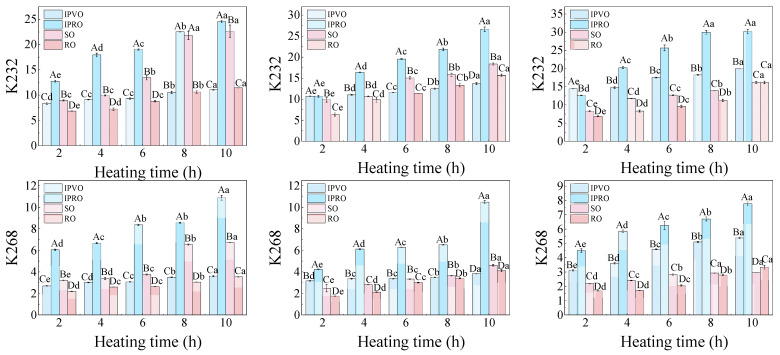
Changes in the values of K232 (**top row**) and K268 (**bottom row**) of IPVO, IPRO, SO and RO during heating at 180 °C (**left**), 200 °C (**middle**), and 220 °C (**right**) for different heating times (2, 4, 6, 8, and 10 h). Different uppercase letters indicate significant differences (*p* < 0.05) between different oil samples at the same heating time and given temperature, while different lowercase letters indicate significant differences (*p* < 0.05) between different heating times for the same oil sample at the same temperature.

Conjugated dienes (CD) and Conjugated trienes (CT) exhibit characteristic absorbance at 232 nm and 268 nm, respectively. The corresponding extinction coefficients (K232 and K268) are therefore commonly used as indicators of lipid oxidation. While CD contributes mainly to K232 and CT contributes to K268, both indices also reflect absorbance from thermally generated compounds such as α- and β-diketones, aldehydes, ketones, and other secondary oxidation products. Thus, CD and CT formation provides useful information on early-stage lipid oxidation, but the changes in K232 and K268 cannot be interpreted as direct measurements of CD or CT [57]. As shown in Figure 6, the K232 values increased with temperature and duration across all four oils, reflecting the accumulation of early-stage oxidation products, including CD, as well as other primary degradation intermediates; The K268 values increased with duration and decreased with temperature indicates that absorbance at 268 nm is not solely related to triene formation. For K232, after 10 h at 220 °C, the values rose from baseline levels of 8.36, 12.73, 8.94, and 6.83 to 19.93, 30.18, 16.16, and 16.17 for IPVO, IPRO, SO, and RO, respectively. A similar trend was observed for K268, with IPVO increasing from 2.71 to 5.38. Unlike K232, the decrease in K268 from 180 °C to 220 °C indicates that the absorbance at 268 nm does not solely reflect CT formation. It may also be due to unsaponifiable compounds (such as sterols, tocopherols, and polyphenols) in *I. polycarbopa* oil initially cause an increase in K268 value, which degrades at higher temperatures, resulting in atypical or decreased absorbance. This phenomenon is consistent with research that secondary components in sesame oil [58], such as sesamin, can affect UV absorption. The pronounced increase in IPRO’s K232 and K268 values compared to IPVO indicates that refining processes may remove natural antioxidants, reducing oxidative stability. Despite similar fatty acid compositions, the slower growth in IPVO suggests effective inhibition of olefinic double-bond migration by residual antioxidants such as polyphenols and tocopherols. The authors of [59] similarly reported that grape seed extracts can suppress the oxidation of linolenic acid and prevent conjugated system formation. Moreover, the greater extent of K232 accumulation relative to K268 in all samples, indicating that the diene structure dominates the early stages of lipid oxidation, while the triene structure or secondary chromophore becomes apparent in later stages and is more susceptible to thermal degradation. This aligns with previous findings showing that K232 formation precedes K268 during lipid oxidation [60]. The high levels of linoleic and linolenic acids (~70%) in IPVO and IPRO also account for their elevated K232 and K268 values over time compared to SO (~61%) and RO (~27%).

These findings reinforce that antioxidant presence, rather than fatty acid saturation alone, dictates the degree of oxidation. IPVO’s performance suggests that minimal processing preserves oxidative resistance, offering benefits in thermally intensive applications.

#### 3.3.6. Polar Compounds

**Figure 7:**
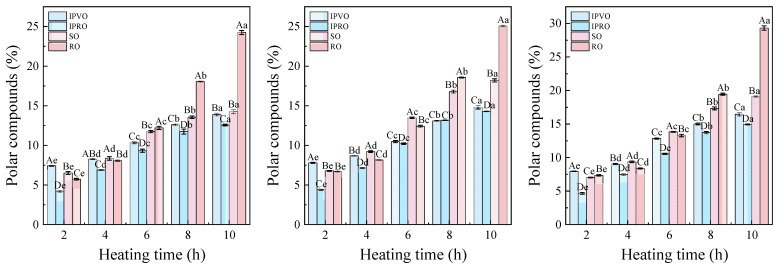
Changes in the values of polar compounds of IPVO, IPRO, SO and RO were observed at different heating times (2, 4, 6, 8, and 10 h) during heating at 180 °C (**left**), 200 °C (**middle**), and 220 °C (**right**). Different uppercase letters indicate significant differences (*p* < 0.05) between different oil samples at the same heating time and given temperature, while different lowercase letters indicate significant differences (*p* < 0.05) between different heating times for the same oil sample at the same temperature.

The threshold value for polar compounds (25–27%) is a key indicator of the degree of oxidative degradation, above which the threshold range is considered to produce toxic and harmful substances [61]. As illustrated in Figure 7, the content of polar compounds in all tested oils—IPVO, IPRO, soybean oil (SO), and rapeseed oil (RO)—increased significantly with both heating temperature and duration. At 180 °C for 10 h, IPVO’s PC content rose from 6.45% to 13.89%, while RO exhibited a more drastic increase from 4.89% to 24.25%. At 220 °C, IPVO reached 16.46%, whereas RO exceeded the safety limit at 29.33%. SO and IPRO also showed significant increases to 19.11% and 14.93%, respectively. The slower increase in IPVO’s PC content compared to other oils suggests superior oxidative stability under thermal stress. This behavior can be attributed to its relatively high antioxidant content, including tocopherols and polyphenols, which interrupt the radical-mediated chain reactions that lead to oxidation and polymerizations [62].

Although RO initially had a lower PC content, its rapid increase and eventual threshold exceedance highlight its vulnerability to thermal degradation, likely due to its very high unsaturated fatty acid content and lack of stabilizing antioxidants. This underscores the importance of balancing unsaturation level with antioxidant preservation for thermal applications [63]. IPVO demonstrated the slowest accumulation of polar compounds during heating, reinforcing its potential as a thermally stable oil for repeated or prolonged high-temperature culinary use.

#### 3.3.7. Fatty Acid Composition and Trans Fatty Acid Composition

Fatty acid composition plays a pivotal role in determining the nutritional value and thermal stability of edible oils. Heating typically leads to the breakdown or isomerization of unsaturated fatty acids, resulting in an increase in saturated fatty acids (SFA) and the formation of undesirable trans fatty acids (TFA), which have been linked to cardiovascular risk [64].

As summarized in Table 5, all four oils—IPVO, IPRO, SO, and RO—showed increases in SFA content and decreases in polyunsaturated fatty acids (PUFA) upon heating from 180 °C to 220 °C. For instance, IPVO’s SFA increased from 17.55% to 17.83%, while PUFA slightly declined from 70.09% to 69.32%. In contrast, RO’s PUFA dropped more significantly from 27.45% to 23.25%, with a corresponding SFA increase from 6.56% to 6.92%. The monounsaturated fatty acid (MUFA) content in IPVO slightly decreased, whereas other oils exhibited increases, indicating that IPVO’s MUFA may be more susceptible to thermal breakdown under prolonged exposure. TFA formation followed a temperature-dependent trend across all oils. The TFA content in IPVO increased from undetectable levels to 0.60% after 10 h at 220 °C, which was lower than IPRO (0.77%), SO (0.87%), and RO (0.70%). Despite its high PUFA content, the lower TFA generation in IPVO suggests that its native antioxidants offer a protective effect by inhibiting double bond isomerization during heating [28,47].

This observation aligns with [65,66], who reported that antioxidant-rich oils exhibit delayed and reduced TFA formation. Thus, IPVO’s composition not only supports nutritional functionality but also ensures better stability under thermal stress. Therefore, IPVO retains a favorable fatty acid profile post-heating with limited trans fatty formation, indicating its suitability for applications requiring thermal processing with minimal nutritional compromise.

## 4. Conclusions

*I. polycarpa* oil has garnered increasing attention due to its high nutritional value. This study found that MVD, minimal impurity levels, and appropriate filtration cycles enhance the oil’s overall quality, resulting in higher oil yield, lower acidity and peroxide values, and greater transparency. Notably, IPVO demonstrated promising oxidative and thermal stability during prolonged heating, as evidenced by its moderate accumulation of oxidation markers such as polar compounds and secondary lipid oxidation products. Compared to IPRO, RO, and SO, IPVO exhibited better resistance to structural degradation and undesirable compound formation under high-temperature conditions. The fatty acid profile of IPVO, rich in polyunsaturated fatty acids, remained relatively stable throughout thermal treatment, and the formation of trans fatty acids was effectively limited. This highlights the potential of IPVO as a functional edible oil that balances nutritional value with technological stability, while its thermal resilience supports applications in high-temperature cooking processes. Collectively, the findings provide a theoretical basis for the high-value utilization of *I. polycarpa* oil. Future research may focus on isolating, identifying, and quantifying bioactive compounds from *I. polycarpa* oil with the aim of developing natural strategies to improve the oxidative stability of *I. polycarpa* oil.

## Figures and Tables

**Table 1:** Processing methods of oil samples obtained under different drying methods (D), impurity contents (I), and filtration cycles (F).

Sample Number	Drying Methods	Impurity Contents/%	Filtration Cycles/Times
D1	MVD	0	1
D2	MD	0	1
D3	FIOD	0	1
D4	ASHPD	0	1
I1	MVD	0	1
I2	MVD	10	1
I3	MVD	20	1
F1	MVD	0	1
F2	MVD	0	3
F3	MVD	0	5

Note: Among them, MD is Microwave Drying, MVD is Microwave Vacuum Drying, FIOD is Far-infrared Oven Drying, ASHPD is Air Source Heat Pump Drying, IC is Impurity Contents, and FC is Filtration Cycles. D1–D4: Four types of drying methods, I1–I3: Three types of impurity content, F1–F3: Three filtration cycles.

**Table 2:** Effect of different drying methods on the quality parameters of IPVO compared with the reference standard LS/T 3258-2018.

Index	MVD	MD	FIOD	ASHPD	LS/T 3258-2018 *Idesia polycarpa* Oil
Transparency (20 °C)	Slightly turbid	Slightly turbid	Turbid	Slightly turbid	Clear, transparent
Smell, taste	*I. polycarpa* oil flavor	*I. polycarpa* oil flavor	Smell of burning	Smell of burning	*I. polycarpa* oil flavor
L* (Lightness value)	58.42 ± 0.01 ^a^	57.75 ± 0.04 ^b^	53.23 ± 0.04 ^d^	53.74 ± 0.04 ^c^	—
a* (Red/Green value)	48.91 ± 0.02 ^c^	52.1 ± 0.01 ^d^	55.83 ± 0.03 ^b^	57.47 ± 0.01 ^a^	—
b* (Yellow/Blue value)	100.22 ± 0.10 ^a^	98.97 ± 0.12 ^b^	91.41 ± 0.11 ^d^	92.33 ± 0.16 ^c^	—
Moisture and Volatiles content (%)	0.04 ± 0.02 ^c^	0.06 ± 0.02 ^bc^	0.07 ± 0.01 ^b^	0.10 ± 0.01 ^a^	≤0.1
Insoluble impurities (%)	0.02 ± 0.00 ^c^	0.09 ± 0.00 ^a^	0 ± 0.00 ^d^	0.03 ± 0.00 ^b^	≤0.05
Acid value (mg KOH/100 g)	1.19 ± 0.02 ^c^	1.45 ± 0.04 ^c^	4.61 ± 0.04 ^a^	3.52 ± 0.07 ^b^	≤3
Peroxide value (meq/kg)	15.76 ± 0.00 ^c^	10.24 ± 0.00 ^d^	23.64 ± 0.00 ^a^	22.06 ± 0.00 ^b^	—
Phospholipid content (mg/g)	0.97 ± 0.05 ^c^	1.30 ± 0.03 ^c^	4.36 ± 0.38 ^a^	2.83 ± 0.58 ^b^	—
Smoke point (°C)	201 ± 1.00 ^a^	197 ± 1.00 ^b^	177.67 ± 1.53 ^d^	181 ± 1.00 ^c^	—

Note: Different letters show statistically significant differences (*p* < 0.05). “—” indicates that the parameter is not specified in the standard.

**Table 3:** Effect of different impurity contents on the quality parameters of IPVO compared with the reference standard LS/T 3258-2018.

Index	IC0%	IC10%	IC20%	LS/T 3258-2018 *Idesia polycarpa* Oil
Transparency (20 °C)	Slightly turbid	Slightly turbid	Slightly turbid	Clear, transparent
Smell, taste	*I. polycarpa* oil flavor	*I. polycarpa* oil flavor	*I. polycarpa* oil flavor	*I. polycarpa* oil flavor
L* (Lightness value)	58.42 ± 0.0115 ^a^	58.15 ± 0.0265 ^b^	55.82 ± 0.0058 ^c^	—
a* (Red/Green value)	52.1 ± 0.01 ^a^	50.33 ± 0 ^b^	48.32 ± 0.0058 ^c^	—
b* (Yellow/Blue value)	100.22 ± 0.101 ^a^	99.69 ± 0.0361 ^b^	95.75 ± 0.1026 ^c^	—
Moisture and volatiles content (%)	0.04 ± 0.02 ^b^	0.1 ± 0.03 ^a^	0.11 ± 0.01 ^a^	≤0.1
Insoluble impurities (%)	0.02 ± 0.00 ^a^	0.02 ± 0.01 ^b^	0.02 ± 0.00 ^a^	≤0.05
Acid value (mg KOH/100 g)	1.19 ± 0.02 ^c^	1.21 ± 0.02 ^b^	1.62 ± 0.00 ^a^	≤3
Peroxide value (meq/kg)	15.76 ± 0.00 ^b^	14.97 ± 0.00 ^c^	16.55 ± 0.00 ^a^	—
Phospholipid content (mg/g)	0.97 ± 0.05 ^b^	1.2 ± 0.08 ^a^	1.17 ± 0.00 ^a^	—
Smoke point (°C)	201.00 ± 1.00 ^a^	200.00 ± 1.00 ^a^	202.00 ± 1.00 ^a^	—

Note: Different letters show statistically significant differences (*p* < 0.05). “—” indicates that the parameter is not specified in the standard.

**Table 4:** Effect of different filtration cycles on the quality parameters of IPVO compared with the reference standard LS/T 3258-2018.

Index	FC1	FC3	FC5	LS/T 3258-2018 *Idesia polycarpa* Oil
Transparency (20 °C)	Slightly turbid	Slightly turbid	Clear, transparent	Clear, transparent
Smell, taste	*I. polycarpa* oil flavor	*I. polycarpa* oil flavor	*I. polycarpa* oil flavor	*I. polycarpa* oil flavor
L* (Lightness value))	58.42 ± 0.0115 ^c^	60.11 ± 0.0115 ^a^	59.58 ± 0.0173 ^b^	—
a* (Red/Green value)	52.1 ± 0.01 ^c^	52.81 ± 0.0058 ^b^	53.21 ± 0.0115 ^a^	—
b* (Yellow/Blue value)	100.22 ± 0.1012 ^c^	103.01 ± 0.0889 ^a^	102.24 ± 0.0896 ^b^	—
Moisture and volatiles content (%)	0.04 ± 0.02 ^b^	0.07 ± 0.00 ^a^	0.11 ± 0.03 ^a^	≤0.1
Insoluble impurities (%)	0.02 ± 0.00 ^a^	0.02 ± 0.00 ^a^	0 ± 0.00 ^b^	≤0.05
Acid value (mg KOH/100 g)	1.19 ± 0.02 ^b^	1.26 ± 0.06 ^a^	1.25 ± 0.04 ^a^	≤3
Peroxide value (meq/kg)	15.76 ± 0.00 ^a^	11.03 ± 0.02 ^b^	11.03 ± 0.0 ^b^	—
Phospholipid content (mg/g)	0.97 ± 0.05 ^b^	1.1 ± 0.03 ^a^	1.07 ± 0.00 ^a^	—
Smoke point (°C)	201.00 ± 1.00 ^a^	197.00 ± 1.00 ^b^	196.67 ± 0.58 ^b^	—

Note: Different letters show statistically significant differences (*p* < 0.05). “—” indicates that the parameter is not specified in the standard.

**Table 5:** Changes in fatty acid composition and trans fatty acid composition of IPVO, IPRO, SO, and RO after 10 h of heating/(area normalized percentage, %).

Fatty Acid	IPVO				IPRO				SO				RO			
	Unh.	180 °C	200 °C	220 °C	Unh.	180 °C	200 °C	220 °C	Unh.	180 °C	200 °C	220 °C	Unh.	180 °C	200 °C	220 °C
C16:0	15.54	15.50	15.61	15.74	15.37	15.82	15.74	15.87	10.67	11.04	11.12	11.51	4.49	4.56	4.57	4.69
C16:1	5.14	5.14	5.18	5.21	5.14	5.26	5.61	5.87	—	—	—	—	—	—	—	—
C18:0	2.01	2.05	2.05	2.09	1.88	1.98	2.31	3.05	3.88	4.07	4.08	4.23	2.07	2.17	2.32	2.23
C18:1	6.36	6.49	6.55	6.60	6.42	6.63	7.08	6.63	27.76	28.79	28.92	29.83	57.80	59.96	59.90	61.33
C18:2	69.23	69.23	69.22	68.67	69.39	68.80	67.55	66.22	51.80	50.43	50.46	49.90	19.12	17.71	17.63	17.79
C18:3	0.86	0.86	0.80	0.66	0.90	0.80	0.75	0.73	5.86	5.35	5.04	3.69	8.33	7.31	6.86	5.46
C22:1	0.94	0.43	0.42	0.43	0.62	0.59	0.52	0.56	—	—	—	—	8.20	8.29	8.72	8.51
t-C18:1	—	—	—	—	—	—	—	—	—	—	—	0.16	—	—	—	0.21
t-C18:2	—	—	0.17	0.60	0.13	0.17	0.28	0.77	0.01	0.27	0.34	0.67	0.00	0.09	0.11	0.24
t-C18:3	—	0.00	0.00	0.00	0.00	0.00	0.00	0.00	0.01	0.05	0.04	0.03	—	—	0.19	0.25
ΣSFA	17.55	17.55	17.66	17.83	17.25	17.80	18.05	18.92	14.58	15.11	15.19	15.74	6.56	6.73	6.89	6.92
ΣMUFA	12.44	12.06	12.15	12.25	12.18	12.49	13.22	13.06	27.76	28.79	28.92	29.83	66.00	68.25	68.63	69.83
ΣPUFA	70.09	70.08	70.02	69.32	70.29	69.59	68.30	66.95	57.66	55.78	55.50	53.59	27.45	25.02	24.49	23.25
ΣTFA	—	0.00	0.17	0.60	0.13	0.17	0.28	0.77	0.02	0.32	0.39	0.87	0.00	0.09	0.31	0.70

Note: Unh, SFA, MUFA, PUFA and TFA denote unheated, saturated fatty acids, monounsaturated fatty acids, polyunsaturated fatty acids, and trans fatty acids, respectively; “—” means not detected.

## Data Availability

The original contributions presented in this study are included in the article/Supplementary Materials. Further inquiries can be directed to the corresponding author.

## Supplementary Materials

The following supporting information can be downloaded at: https://www.mdpi.com/article/10.3390/foods14244210/s1, Table S1: Main effect analysis table.
