# Supplementary material for: Pretreatment and Thermal Stability of Idesia polycarpa Virgin Oil
Source: Foods. 2025 Dec 8;14(24):4210. doi: 10.3390/foods14244210 (PMC12731739; doi:10.3390/foods14244210)
Supplement: Supplementary file 1 [file foods-14-04210-s001.zip › foods-3995264-supplementary.pdf]

Supplementary Table S1. Main effect analysis table.

Source of Variation	Sum of Squares	df	Mean Square	F	*p*
Intercept	5006.71	1	5006.711	24365.927	**
Drying methods	47.047	3	15.682	76.321	**
Impurity content	1.379	2	0.689	3.355	**

Note: R^2 = 0.946 (Adjusted R^2 = 0.928); ** $p < 0.01$, df = degrees of freedom.
